# Association between attitude towards death and spiritual care competence of Chinese oncology nurses: a cross-sectional study

**DOI:** 10.1186/s12904-021-00846-8

**Published:** 2021-09-29

**Authors:** Liujin Li, Jingmin Lv, Lingling Zhang, Yalan Song, Ying Zhou, Jiaxian Liu

**Affiliations:** 1 School of Nursing, Guangzhou Medical University, 195 Dongfeng West Road, Yuexiu District, Guangzhou, P.R. China; 2 Nursing Department, Affiliated Cancer Hospital & Institute of Guangzhou Medical University, Guangzhou, China

**Keywords:** Spiritual competence, Oncology, Nurses, Attitudes towards death

## Abstract

**Backgrounds:**

An understanding of the oncology nurse spiritual care competence would help nurse managers recognize weakness in spiritual practice and improve the quality of spiritual care. But the relationship between attitude towards death and spiritual care competence is unknown.

**Methods:**

We recruited 326 nurses from hospitals in Guangzhou, China. The nurses completed the Chinese Spiritual Care Competence Scale and the Chinese Death Attitude Profile-Revised questionnaires.

**Results:**

The total score of spiritual care competence was 61.62 ± 16.10. And the lowest score of attitude towards death was for *escape acceptance*, 2.64 ± 0.82. Factors associated with nurse spiritual care competence were work department, whether trained in spiritual care, *approaching acceptance*, and *escaping acceptance* of attitude towards death.

**Conclusion:**

Nurses need to perfect their spiritual care competence and establish positive attitudes towards death.

## Background

Spirituality is viewed as an essence, a driving force, and a self-supporting resource [1]. Spiritual care is the recognition and response to human spiritual needs. Spiritual care meets the needs of life meaning, self-worth, expressing oneself, and faith support [2]. Spiritual care is a required element of holistic health care. A recent concept analysis of spiritual care suggested that spiritual care includes healing presence, therapeutic use of self, intuitive sense, exploration of the spiritual perspective, patient-centeredness, meaning-centered therapeutic intervention, and creation of a spiritually nurturing environment [3]. Spiritual care competence (SCC) refers to an ability to assess and identify patient spiritual needs and implement appropriate interventions to promote patient spiritual health. SCC requires knowledge, attitudes, and skills of spiritual care [4].

Cancer patients generally have a high level of spiritual need [5–7]. Höcker et al. found that most cancer patients indicated at least one spiritual need [5]. Spiritual needs increase drastically after a diagnosis of cancer [6, 7] because people usually associate cancer with death, and they are vulnerable to depression and anxiety. In addition, the spiritual issue also affects a patient’s cooperation with treatment and rehabilitation. Therefore cancer patients’ spiritual issues should receive more attention. Spiritual care for cancer patients can improve negative mental states such as anxiety and depression and relieve fatigue and pain. In addition, spiritual care can help patients contemplate the meaning of life and gain peace of mind, so that they can face disease and death [8–10]. Nurses, the most accessible healthcare providers for patients, have responsibilities in spiritual support. Somayeh et al. and Nkomo found that clinical nurses could not adequately assess or identify patient spiritual needs, and nurses lacked sufficient awareness of patient spiritual problems [11, 12]. Thus, it is essential to improve the SCC of oncology nurses.

There are some studies on spiritual care competence of nurses, but little is known about the spiritual care competence of nurses in non-religious contexts. Investigators have assessed the relationship between spiritual care competence and nurse personal characteristics; findings from these studies could help to understand how to effectively improve spiritual care competence. Studies have shown that nurse spiritual care competence is related to the perception of spirituality/spiritual care [13] and to personality characteristics [14, 15] such as spiritual well-being [16] and self-efficacy [17]. But little is known about the relationship of nurse attitude towards death and spiritual care competence.

Attitude about death is a stable and evaluative psychological tendency when individuals face death environment. Attitude towards death encompasses *fear of death*, *death avoidance*, *natural acceptance*, *approach acceptance*, and *escape acceptance* [18]. Ay and Öz [19] showed that oncology nurses often have negative feelings about death, such as fear and anxiety. These feelings could directly affect the mental health of nurses themselves and the quality of nursing care. Schroeder et al. reported that nurses with positive attitudes towards death had a positive influence on the care of their patients [20]. Akdeniz found that nurse attitude towards death could affect their spirituality and spiritual perception, which was an important factor that affected their SCC [21]. A high degree of nurse negative attitudes towards death impaired their level of perceiving spirituality and spiritual care. And nurses with negative attitudes towards death had difficulty providing spiritual care [22, 23]. However, the relationship between attitude towards death and nurse SCC has not been reported for the Chinese cultural background. Thus, it is essential to assess the effects of attitude towards death on nurse spiritual care competence to provide a means to improve nurse spiritual care competence.

## Methods

### Aim

The aim of this study was to establish oncology nurse spiritual care competence and its relationship with the attitude toward death and personal characteristics. The specific purposes were the following:

1. To measure the spiritual care competence of Chinese oncology nurses.
2. To document Chinese oncology nurse attitudes towards death.
3. To identify factors that affect nurse spiritual care competence.
4. To assess the relationship between nurse spiritual care competence and attitude towards death.

### Study design

A descriptive cross-sectional study using a field questionnaire and online survey methods was conducted in Guangzhou, Guangdong province, China. The research conformed to the Strengthening the Reporting of Observational Studies in Epidemiology (STROBE) Statement: Guidelines for Reporting Observational Studies. All methods were conducted in accordance with relevant guidelines and regulations. The theory of this study is based on Bandura’s social cognitive theory.

### Participants

The principle of multifactor analysis requires a sample size 5-10 times the number of variables. The number of variables in this study was 21; thus, the sample size was about 21*(5-10) = 105-210. The sample size was increased by 20%, to 126-252, to protect against possible data loss or invalid data. Convenience sampling was used to select nurses from a cancer hospital and four Frist-class Hospital at Grade 3 in Guangdong Province, China, from December 2019 to March 2020. To be eligible, clinical nurses had to have been engaged in clinical tumor-related nursing work for more than one year; nursing students, nurse interns, and nursing managers were excluded. Three hundred twenty-six oncology nurses volunteered to participate in the research.

### Demographics and professional experiences

We collected participant demographics including sex, age, marital status, religious belief, the highest degree of nursing education, professional experience such as title, years of nursing experience, working unit, experience of caring for terminally ill patients, experience caring for a terminally ill family member, whether training was received for perceptions of competence in delivering, and whether a family member or friend was a cancer patient.

### Spiritual care competence scale

Van Leeuwen et al. [24] developed the Spiritual Care Competence Scale (SCCS), a self-reporting scale to measure nurse spiritual care competence. The questionnaire contains 27 items, consisting of six core domains of spiritual care-related nursing competencies (*assessment and implementation of spiritual care, professionalization and improving quality of spiritual care, personal support and patient counseling, referral to professionals, attitude towards patient’s spirituality and communication*). All items are scored on a 5-point scale from completely disagree to fully agree, scoring from 1 to 5. The original scale was validated with nursing students and showed sound reliability and validity [24]. Di et al. [25] revised the Chinese version of the SCCS (SCCS-C); they translated and back-translated the scale using Brisling’s translation model after approval from the original author. The final SCCS-C is composed of 22 items and the 6 dimensions of the original scale. For a better understanding of the Chinese population, the revised scale specifies “never, seldom, sometimes, often, and always” as possible answers, with scoring from 1 to 5. The range of score is 22–110. A high total score indicates a participant’s high level of spiritual care competence. The psychological metrics were validated with 528 nurses in mainland China. The content validity index of the SCCS-C was 0.98. The construct validity was tested by confirmatory factor analysis. The six-factor structure fit the data well, which accounted for 77.43% of the total variance. The *Cronbach’s α* for the SCCS-C was 0.974 and 0.902 ~ 0.956 for different dimensions, which indicated satisfactory reliability [25]. In this study, reliability was examined by analysis of *Cronbach’s a* (=0.952), an estimate of internal consistency. Sampling adequacy was confirmed by Kaiser-Meyer-Olkin measure (=0.954, *p* < .001).

### Death attitude profile-revised

The Death Attitude Profile-Revised (DAP-R) was developed by Wong et al. (1994) [26] to evaluate attitudes towards death, and the validity and reliability of the Chinese version was assessed by Tang Lu et al. [18]. The scale is multidimensional with 32 items and 5 subgroups: *fear of death, death avoidance, neutral acceptance, approach acceptance and escape acceptance*, and which dimension has the highest score indicates that the participants tend towards the attitude of death represented by that dimension. The scale is of the Likert-5 type and rated as 1-strongly disagree to 5-strongly agree. A total score is 32 ~ 160. The *Cronbach’s α* for DAP-R was 0.80 [18]. In this study, the Kaiser- Meyer- Olkin for DAP-R was 0.954 (*p* < .001), the *Cronbach’s α* was 0.898 and 0.671 ~ 0.882 for different dimensions, which indicated satisfactory reliability.

### Procedure

From December 2019 to January 2020, data were collected by field questionnaire and online survey from nurses at one tumor specialist hospital in Guangzhou. Completing the survey was voluntary. About 10-15 min were required to complete the questionnaire. One hundred questionnaires were distributed, and 87 valid questionnaires were returned.

Because of the SARS-CoV-2 pandemic, we stopped taking questionnaires at hospitals and continued to collect data online. The online survey was constructed with Wen Juan Xing (www.wjx.cn), an electronic data collection tool. The link to the questionnaire was posted on the Internet and sent to one tumor specialist hospital and three general hospitals in Guangzhou. These hospitals were invited to share the survey link with their oncology nurses. Completing the survey was voluntary. The survey link was available from February 2020 to March 2020. Data collection lasted 10–15 min. Each IP address and account could answer the survey only once to avoid duplication. To ensure the quality of the data, we eliminated questionnaires that were completed in a short time (< 3 min) or a long period (> 30 min). Finally, 326 valid questionnaires were collected.

### Statistical analysis

The data collected online were transferred to statistical software SPSS 23.0 for analysis. Categorical variable data (participant demographics and professional experiences) were presented as numbers and percent, whereas continuous variables (SCC, attitude towards death) were expressed as means and standard deviation (SD). *Spearman* correlation analysis was conducted to detect a relationship between SCC and attitude towards death. Multiple linear regression analysis was performed to determine the factors associated with SCC. The total SCC score was set as the dependent variable, whereas demographics, professional experience, and dimensions of DAP-R were independent variables. A *p* value of < 0.05 was considered statistically significant.

### Ethical considerations

The Ethics Committee of Guangzhou Medical University approved this study. Participants provided informed consent prior to participation.

## Results

### Demographics and professional experience

Of 326 participants, 95.4% (*n* = 311) were women. Most were less than 35 years old. Ninety-five percent (*n* = 308) were non-religious, and 39% (*n* = 127) were unmarried. Sixteen percent of the participants (*n* = 52) received their highest degree of nursing education below bachelor, and 80.44% (*n* = 262) achieved a bachelor’s degree. Most nurses were of middle title. Thirty-eight percent (*n* = 124) were working in oncology internal medicine, and 30.7% (*n* = 100) were working in surgical oncology. A little more than half of the nurses had more than six years of working experience (63.8%) and had experience in caring for terminally ill patients (77.0%). Forty-three percent (*n* = 139) had never participated in the care of a family member or friend. Fifty-seven percent (*n* = 187) stated that they did not receive any training related to spiritual care. Table 1 presents additional demographic information.

**Table 1:** Demographics and professional experience of oncology nurses (*n* = 326)

Variable		n	%
Sex	Male	15	4.60
	Female	311	95.40
Age	≤25 year	50	15.30
	26-35 year	198	60.70
	36-45 year	62	19.00
	≥46 year	16	4.90
Religion	No	308	94.50
	Yes	18	5.50
Highest degree of nursing education	Junior college	52	16.00
	Bachelor’s degree	262	80.40
	Master’s degree	12	3.70
Marital status	Single	127	39.00
	Married/divorced	199	61.00
Nursing clinical ladder	N0/N1	68	20.90
	N2	161	49.40
	N3	92	28.20
	≥N4	5	1.50
Working experience	≤3 year	48	14.70
	4-6 year	70	21.50
	7-9 year	78	23.90
	10-12 year	52	16.00
	≥13 year	78	23.90
Working unit	Oncology internal medicine	124	38.00
	Oncology surgical	100	30.70
	Other	102	31.30
Trained in spiritual care?	No	269	82.50
	Yes	57	17.50
Participated in patient death care?	No	75	23.00
	Yes	251	77.00
Participated in family or friend death care?	No	187	57.40
	Yes	139	42.60
Family or friend who is/was a cancer patient?	No	172	52.80
	Yes	154	47.20

### SCC

As presented in Table 2, the scores for the total scale and subscales of SCCS-C described nurse spiritual care competence. The total score of SCCS-C was 61.12 ± 16.10, ranging from 22 to 110. The highest score on competence was for *communication,* 3.64 ± 1.00, and the participants had the lowest score for competence on *referral to professionals*, 2.27 ± 0.93.

**Table 2:** Mean scores of SCCS and DAP-R (*n* = 326)

Variable	Score	Range	Item average score
SCCS total	61.62(16.10)	22-110	2.80 ± 0.73
Assessment and implementation of spiritual care	10.62(3.47)	4-20	2.65 ± 0.87
Professionalization and improving the quality of spiritual care	13.17(4.25)	5-25	2.63 ± 0.85
Personal support and counselling of patients	12.29(4.24)	5-25	2.46 ± 0.85
Referral to professionals	4.54(1.85)	2-10	2.27 ± 0.93
Attitude towards patient spirituality	13.71(4.26)	4-20	3.43 ± 1.07
Communication	7.29(1.99)	2-10	3.64 ± 1.00
DAP-R			
Fear of death	20.11(4.88)	7-35	2.87 ± 0.70
Death avoidance	14.86(3.76)	5-25	2.97 ± 0.75
Neutral acceptance	19.14(3.12)	5-25	3.83 ± 0.62
Approach acceptance	27.67(6.65)	10-50	2.77 ± 0.67
Escape acceptance	13.19(4.10)	5-25	2.64 ± 0.82

Note: *SCCS* Spiritual Care Competence Scale; *DAP-R* Death Attitude Profile-Revised Scale

### Attitude towards death

Table 2 lists the scores for the subscales of DAP-R that described participant attitudes towards death. The highest score on DAP-R was for *neutral acceptance,* 3.83 ± 0.62, and the lowest score was *escape acceptance,* 2.64 ± 0.82. The participants had scores of 2.87 ± 0.70 on *fear of death*, 2.77 ± 0.67 on *death avoidance*, and 2.77 ± 0.67 on *approach acceptance.*

### Relationship between **SCC** and attitude towards death

The *Spearman* correlation (Table 3) showed that SCCS was related with *Fear of death, Death avoidance, Neutral acceptance,* and *Approach acceptance*, with the correlation coefficients ranging from 0.109 to 0.198. This result indicates that the SCC is weakly and positively correlated to the attitude towards death.

**Table 3:** Relationship between spiritual care competence and attitude towards death (n = 326)

Variable	Fear of death	Death avoidance	Neutral acceptance	Approach acceptance	Escape acceptance
SCCS total	.109*	.181**	.143**	.198**	0.04

Note: *SCCS* Spiritual Care Competence Scale

*Significance at *p* < 0.01; **Significance at *p* < 0.05

### Factors associated with **SCC**

Table 4 shows the results of regression analysis. The linear regression equations fit the data well (F = 4.985, *p* < .001), which explained 18.1% of the variance. The absolute value of the standardized regression coefficient showed a significant association with the dependent variable. The associations between the SCC and demographics, professional experience, and dimensions of attitude towards death were as follows: work department, whether trained on spiritual care, *approaching acceptance,* and *escaping acceptance*.

**TABLE 4:** Results of multiple linear regression analysis of associated factors of spiritual care competence (n = 326)

Model	Unstandardized Coefficients		Standardised Coefficients	t	Sig.	95.0% Confidence Interval for B	
	B	Std.Error	Beta			Lower Bound	Upper Bound
Constant	51.463	11.656		4.415	0**	28.527	74.4
Gender	−3.355	4.024	−0.044	−0.834	0.405	−11.274	4.563
Age	−3.516	1.876	−0.158	−1.874	0.062	−7.208	0.176
Religion	−4.637	3.634	−0.066	−1.276	0.203	−11.787	2.514
Education	0.066	2.192	0.002	0.03	0.976	−4.248	4.379
Marital status	0.563	2.127	0.017	0.265	0.792	−3.623	4.749
Working experience in years	0.135	1.108	0.012	0.122	0.903	−2.046	2.316
Nursing clinical ladder	0.104	1.773	0.005	0.059	0.953	−3.384	3.592
Oncology internal	3.477	1.991	0.105	1.746	0.082	−0.441	7.395
Oncology surgical	5.983	2.092	0.172	2.86	0.005*	1.867	10.1
Have been trained on spiritual care.	12.286	2.212	0.29	5.555	0**	7.934	16.638
Have been participated in patients’ death care	1.632	2.104	0.043	0.776	0.439	−2.508	5.772
Have been participated in family or friends’ death care	1.22	1.811	0.038	0.673	0.501	−2.344	4.783
Have some family or friends who is/was cancer patient	0.784	1.735	0.024	0.452	0.652	−2.631	4.199
Fear of death	−0.104	0.224	−0.031	−0.463	0.644	−0.545	0.337
Death avoidance	0.436	0.296	0.102	1.475	0.141	−0.146	1.019
Neutral acceptance	0.126	0.281	0.024	0.449	0.654	−0.427	0.68
Approach acceptance	0.777	0.187	0.321	4.154	0**	0.409	1.145
Escape acceptance	−0.632	0.284	−0.161	−2.222	0.027*	−1.191	−0.072

Note: The overall model: F(22, 110) = 4.985, *p* = .000; R2 = .226; adjusted R2 = .181

*Significance at *p* < 0.01; **Significance at *p* < 0.05

## Discussion

### The situation of oncology nurse SCC

The total score of SCCS-C of oncology nurses in Guangzhou was 61.12 ± 16.10, which was at a medium level and slightly lower than the score reported by Yang et al. [15]. The difference in scores may be related to differences in the nurse populations. Moreover, the SCC scores in our study were significantly lower than those reported by Petersen et al. [27], who investigated 112 pediatric oncology nurses (98.30 ± 14.05) scores on SCCS [27], and Attard et al. [28] who investigated nurses (105.73 ± 14.053) and midwives (104.37 ± 8.999). The reason for this difference may be due to differences in religious, cultural, educational, and spiritual aspects. Spiritual care is highly valued abroad, and theoretical training and clinical practice in spiritual caring for terminally ill patients are more mature external to China. The American Clinical Oncology Association [29] palliative treatment guidelines include spiritual care in the basic content of cancer patient care. Spiritual nursing in China began relatively late. At present, there is a lack of a unified spiritual concept, systematic intervention, and an independent care model [4, 30, 31]. Chinese clinical nurses have a different understanding of spirituality. Some even consider spirituality a matter of personal privacy or they equate it with religion [32, 33]. Therefore, it is necessary to define the connotation of spirituality and spiritual care, to increase nurse recognition of spirituality, to raise their recognition of spiritual distress, and to promote the implementation of spiritual care.

*Communication* and *attitude towards patient spirituality* of SCC had the highest scores in this study; the scores were similar to other studies [13–15, 34, 35]. The results showed that nurses were confident about their communication with patients on spiritual issues, and nurses respected patient spirituality. We found that nurses received the lowest score on *referral to professional*, which was different from the results of Azarsa et al. [36]. *Referral to professional* refers to transferring or recommending relevant spiritual care professionals or institutions to patients who have severe spiritual distress, so that patients can get timely and effective intervention. There are many spiritual nursing professional care teams outside of China, and healthcare providers have abundant resources to refer patients [27, 37]. Because of cultural differences between China and the West, and a lack of Chinese standard procedures of spiritual care, healthcare providers are not clear about their roles in spiritual support and the means of referral. These factors hinder nurses from referring patients to other professionals or organizations for more effective help. Thus, additional research is needed to assess the appropriate spiritual care mode for Chinese patients, determine the roles of healthcare providers in delivering spiritual care, and develop a professional spiritual support system. In addition, government should provide appropriate support.

### The influencing factors of oncology nurse SCC

We found that, compared with oncology internal medicine and other departments, the SCC of oncology surgical nurses was relatively high. The high SCC may be related to the short hospitalization period of tumor surgery patients and their more limited spiritual needs [38, 39].

We also found that nurses who been trained in spiritual care showed higher degrees of SCC, which was similar to findings by Yang et al. [15] and Hsieh et al. [13]. This result indicated that spiritual care training could enhance SCC. However, only 17.5% participants in our study received spiritual care training, lower than the 19.2% and 57.9% reported by Chen et al. [34] and Yang et al. [15], respectively. Thus, the curriculum and learning requirements are lacking in China. At present, Chinese medical colleges and universities have not unified spiritual care education content, and they lack appropriate spiritual education resources. This situation means that it is necessary to combine humanistic care to conduct spiritual education and improve the training system of spiritual care [40–42]. Nursing administrators also need to improve oncology nurse cognition and attention to spiritual care, provide more training and education opportunities, and improve nurse SCC by expert theoretical teaching, spiritual care case discussion, and sharing of successful care experiences from departments with high SCC.

In our study, the *natural acceptance* had the highest score, which was similar to other reports [43, 44]. In addition, we found that attitude towards death was positively associated with nurse SCC. Oncology nurses held an objective and positive acceptance of death, which was akin to results reported by Du et al. and Han et al. [43, 45]. The high scores for *death escape* and *death fear* indicated that some nurses had a negative feeling about death, which may have been related to the high frequency of death care and impairment of nurse mental health. Nursing educators should attach importance to death education, improve the death education system, increase death education in continuing education, enhance nurse positive view of death, and improve nursing quality.

### Effects of death attitude of oncology nurses on their SCC

A strong negative attitude towards death led to low levels of perceiving spirituality and spiritual care [21]. Investigators have pointed out that a negative view of death affects nurse cognition of spiritual and spiritual care, and spiritual care cognition is an important factor that affects nurse SCC [13, 46–48]. However, there are no reports of whether different death attitudes are related to spiritual care. We found a positive correlation between the death *approach acceptance* and nurse SCC (*P* < 0.001). Nurses who had *approach acceptance* (an attitude that accepts death optimistically and regards death as part of life or as a way to a happy next life) had higher spiritual care competence. The reason for this relationship may be that nurses with a heart-felt positive acceptance of death can better detect the spiritual distress of cancer patients and provide spiritual care. Conversely, after looking at death positively, nurses will improve their spiritual care in the process of nursing. They will provide spiritual care by listening to companionship and soothing resonance to convey a positive view of life and death in the process of caring for cancer patients, to help cancer patients treat and face death correctly and find inner peace and comfort to maintain their spiritual health.

Furthermore, we found that the *escape acceptance* was negatively correlated with nurse SCC (*P* < 0.05). This negative correlation was possible because nurses with negative evasive attitudes to death were unable to detect the spiritual distress of cancer patients, their understanding of spirituality was not deep, and they were unable to perform spiritual care and relieve patient spiritual distress. Hsieh et al. [13] and Shi et al. [47] pointed out that nurse cognition and spiritual perception can affect the quality of spiritual care. Death attitude may have affected nurse SCC by the intermediary of spiritual cognition and perception, but the specific mechanism needs further study.

We found that the death attitude of oncology nurses was not optimistic. Although most participants had natural acceptance, there were still some negative factors such as death fear and escape. Nursing administrators should recognize the death attitude of oncology nurses, promote oncology nurses to approach death by improving their self-acceptance level, and construct effective death and spiritual education models. Managers should craft strategies to enhance positive death attitude of oncology nurses, improve their SCC, improve the quality of spiritual care, and better maintain the spiritual health of cancer patients.

### Strengths and limitations

The strengths of our study are the following factors. First, we measured the relationship between spiritual care competence and attitude towards death, which is scarcely reported. Second, to our best knowledge, there are few reports on spiritual care competence in mainland Chinese. Our study will contribute to understanding of spiritual care competence among Chinese oncology nurses. Third, a study of a Chinese population was needed because of important differences in culture and health care systems between mainland China and elsewhere and because of the ever-increasing importance of spiritual care.

Some limitations of our study need to be acknowledged. Although the sample size was sufficient, the participants were oncology nurses from a single province. Further studies are needed to assess the status in other provinces and enroll more participants. We used a cross-sectional survey that could not report changes in nurse spiritual care with time. In addition, SCCS is a self-report instrument that may have response bias because participants tend to give acceptable answers that differ from their actual level of competence.

## Conclusion

Oncology nurses showed different levels of competence in various dimensions of spiritual care. At present, the incidence of cancer is high, and most cancer patients have spiritual issues. Spiritual care is becoming increasingly important in oncology nursing. It is imperative to enhance oncology nurse competence to address the spiritual needs of patients and improve patients quality of life. Nurses also need to perfect their spiritual care competence by different ways; establishing a positive attitude towards death could be an option.

### Implication for practice

Because of inadequate spiritual care competence in China, patient spiritual issues may be ignored or under-recognized, especially for cancer patients who have more spiritual issues that other patients. Nurse managers need to enhance oncology nurse competence to address the spiritual needs of patients. Our study revealed deficiencies in oncology nurse spiritual care competence. Therefore, nurse managers know which areas of the nurse spiritual care competence need strengthening. There is a need for appropriate strategies that emphasize the importance of spiritual care by nurses and enhance nurse spiritual care competence, such as training and clinical practice on spiritual care. In addition, enhancing one positive attitude towards death, such as approach acceptance, is likely to improve spiritual care competence.

## Data Availability

The data used during this study are available from the corresponding authors on reasonable request.

## Abbreviations

SCC: Spiritual care competence
SCCS: Spiritual Care Competence Scale
SCCS-C: Chinese version of the SCCS
DAP-R: Death Attitude Profile-Revised
SD: Standard deviation
